# Longitudinal molecular microbial analysis of influenza-like illness in New York City, may 2009 through may 2010

**DOI:** 10.1186/1743-422X-8-288

**Published:** 2011-06-09

**Authors:** Rafal Tokarz, Vishal Kapoor, Winfred Wu, Joseph Lurio, Komal Jain, Farzad Mostashari, Thomas Briese, W Ian Lipkin

**Affiliations:** 1 Center for Infection and Immunity, Mailman School of Public Health, Columbia University, USA; 2 The New York City Department of Health and Mental Hygiene, New York, USA; 3 The Institute for Family Health, New York, New York, USA

## Abstract

**Background:**

We performed a longitudinal study of viral etiology in samples collected in New York City during May 2009 to May 2010 from outpatients with fever or respiratory disease symptoms in the context of a pilot respiratory virus surveillance system.

**Methods:**

Samples were assessed for the presence of 13 viruses, including influenza A virus, by MassTag PCR.

**Results:**

At least one virus was detected in 52% of 940 samples analyzed, with 3% showing co-infections. The most frequently detected agents were rhinoviruses and influenza A, all representing the 2009 pandemic H1N1 strain. The incidence of influenza H1N1-positive samples was highest in late spring 2009, followed by a decline in summer and early fall, when rhinovirus infections became predominant before H1N1 reemerged in winter. Our study also identified a focal outbreak of enterovirus 68 in the early fall of 2009.

**Conclusion:**

MassTag multiplex PCR affords opportunities to track the epidemiology of infectious diseases and may guide clinicians and public health practitioners in influenza-like illness and outbreak management. Nonetheless, a substantial proportion of influenza-like illness remains unexplained underscoring the need for additional platforms.

## Background

In 2009 an influenza pandemic was precipitated by the emergence of a novel H1N1 influenza A virus that represented a reassortant of previously circulating avian, human and swine viruses [1-5]. The first cases were reported in March and April 2009, initially from the Veracruz region of Mexico, immediately followed by reports from California and Texas in the United States (US) [6,7]. By late April 2009 over half of the total confirmed US cases were from New York City (NYC) [8]. The virus quickly spread globally, with over 17,000 cases reported from 62 countries by June 1, 2009. In response to the outbreak, the NYC Department of Health and Mental Hygiene (DOHMH) implemented an enhanced citywide influenza surveillance program focusing on cases of severe influenza and influenza-like illness (ILI) in hospitals [9]. To enable surveillance in ambulatory care settings, samples from patients presenting with fever or respiratory illness were collected in nine community health care centers over a period of thirteen months, and analyzed for respiratory viruses by MassTag PCR, a multiplex molecular platform for diagnostic microbiology [10-15].

## Methods

Nasopharyngeal (NP) swabs were collected at 9 New York City community health centers of the Institute for Family Health between May 29, 2009 and May 27, 2010 from patients with fever (>37.7°C) or symptoms of respiratory infection. Peak numbers were collected within the first two months of the pandemic and again in fall and winter, coinciding with the seasonality of ILI in the northern hemisphere. The study protocol was approved by the Institutional Review Boards of the DOHMH (IRB#09-031) and Institute for Family Health and consent was obtained from all patients. Samples were tested by MassTag PCR [10] for the presence of 13 viruses including influenza A (FLUAV), influenza B (FLUBV), human rhinoviruses (HRV), human enteroviruses (HEV), human metapneumovirus (HMPV), human parainfluenza virus (HPIV) 1-4, human coronaviruses (HCoV) 229E and OC43, and respiratory syncytial virus (RSV) A and B. All positive samples were re-amplified by singleplex PCR for sequence-based typing.

## Results

**Figure 1:**
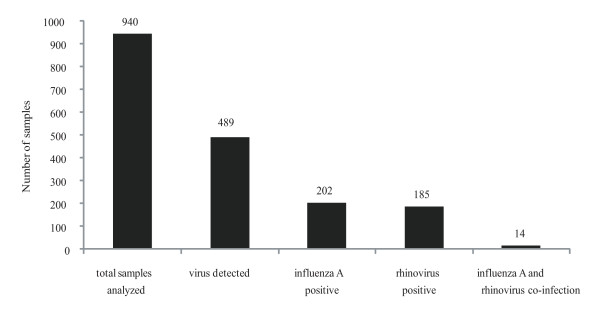
**Summary of virus infections and co-infections with influenza A virus and human rhinovirus**.

**Figure 2:**
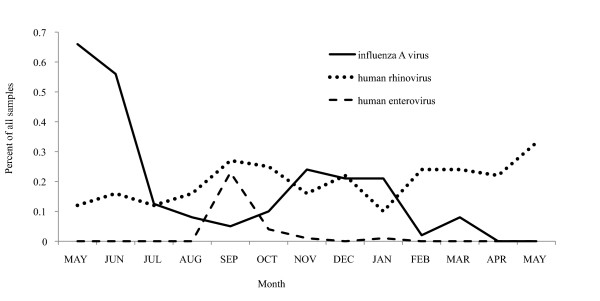
**Monthly incidence of influenza A virus, human rhinovirus and human enterovirus**. Each data point is shown as a percentage of all samples screened during that month.

**Figure 3:**
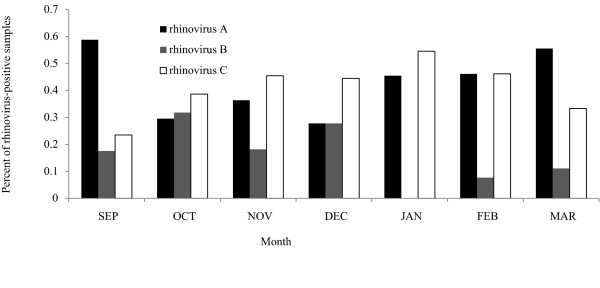
**Distribution of human rhinovirus A, B, and C detected in samples from September 2009 to March 2010**. Data from May-Aug 2009 and Apr-May 2010 is not shown due to low number of rhinovirus-positive samples. Each bar represents the percentage of all sequenced rhinoviruses detected in that month.

A total of 940 samples were analyzed (Table 1). At least one agent was detected in 489 samples (52%). The viruses most commonly detected were influenza A virus and rhinoviruses at 202 (21%) and 185 (20%), respectively (Figure 1). All influenza A viruses were the 2009 H1N1 pandemic strain (A/H1N1/09) as indicated by sequence analysis; one sample was positive for influenza B virus. Influenza A virus was detected in the majority of samples collected from May through June 2009, and again during November 2009 to January 2010, whereas rhinoviruses predominated during August through October 2009 and February to May 2010, when influenza A virus activity was low (Figure 2). The frequency of detection of human rhinovirus A, B and C species varied over the sampling interval (Figure 3). Infections were more common with rhinovirus A (41%) and rhinovirus C (39%) than rhinovirus B (20%). In September 2009, 14 samples (23%) were enterovirus-positive. Sequencing of a 500 base-pair fragment in the VP4/2 gene region indicated that all of these enterovirus-positive samples represented a single strain of enterovirus 68. The number of samples positive for this virus declined to six in October. Only four additional enterovirus-positive samples were obtained during the rest of the study; three were coxsackievirus A2 and one coxsackievirus A6. A total of 30 (3%) of 940 virus samples had evidence of co-infection with two or more viruses; 14 of these showed co-infection with influenza virus and rhinovirus (Figure 1). Collectively, influenza viruses and rhinoviruses accounted for 74% of all viruses detected in our sample set. Other viruses detected, in order of decreasing frequency, included HMPV (3%), RSV-A (2%), HPIV-3 (2%), HCoV-OC43 (1%), HPIV-1 (1%), RSV-B (1%), HPIV-2 (1%), HCoV-229E (1%), and HPIV-4 (<1%)(Table 1). The peak activity for HCoV-OC43 occurred during late fall/early winter months, overlapping with the seasonal peak of RSV-A and -B, while the detection of HMVP, HPIV-A and -3 did not show a significant seasonality.

**Table 1:** Distribution of agents detected in respiratory samples in New York City, May 2009 through May 2010

	Total samples	FLUAV	FLUBV	HEV	HRV	HCoV-OC43	HCoV-229E	HMPV	HPIV-1	HPIV-2	HPIV-3	HPIV-4	RSVA	RSVB
May	24	16 (66%)	0	0	3 (12%)	0	0	1 (4%)	0	0	0	0	0	0

Jun	116	65 (56%)	0	0	18 (16%)	0	1 (1%)	1 (1%)	2 (2%)	1 (1%)	5 (4%)	0	0	0

Jul	16	2 (12%)	0	0	2 (12%)	0	0	0	0	0	4 (25%)	0	0	0

Aug	12	1 (8%)	0	0	2 (16%)	0	0	0	0	0	0	1 (8%)	0	0

Sep	62	3 (5%)	0	14 (23%)	17 (27%)	0	0	1 (2%)	1 (2%)	0	0	0	0	0

Oct	179	18 (10%)	0	8 (4%)	44 (25%)	2 (1%)	0	0	3 (2%)	2 (1%)	1 (<1%)	1 (<1%)	0	0

Nov	137	33 (24%)	0	1 (1%)	22 (16%)	1(1%)	0	2 (1%)	1 (1%)	1 (1%)	0	0	5 (4%)	0

Dec	182	38 (21%)	0	0	40 (22%)	8 (4%)	1 (<1%)	1 (<1%)	1 (<1%)	1 (<1%)	0	0	11 (6%)	2 (1%)

Jan	106	22 (21%)	0	1 (1%)	11 (10%)	2 (2%)		3 (3%)	1 (1%)	1 (1%)	4 (4%)	0	4 (4%)	3 (3%)

Feb	54	1 (2%)	0	0	13 (24%)	0	2 (4%)	12 (22%)	0	0	0	0	0	2(4%)

Mar	37	3 (8%)	0	0	9 (24%)	0	1 (3%)	1 (3%)	1 (3%)	0	1 (3%)	0	0	0

Apr	9	0	1 (10%)	0	2 (22%)	0	1 (10%)	1 (10%)	0	0	1 (10%)	0	0	0

May	6	0	0	0	2 (33%)	0	0	0	0	0	2 (33%)	0	0	0

total	940	202 (21%)	1 (<1%)	24 (3%)	185 (20%)	13 (1%)	6 (1%)	24 (3%)	10 (1%)	6 (1%)	18 (2%)	2 (<1%)	20 (2%)	7 (1%)

## Discussion

Our analysis of respiratory samples from NYC collected during the first year of the A/H1N1/09 outbreak indicates that the novel recombinant virus had quickly established itself as the primary circulating influenza virus in the city. Notably, all influenza A virus-positive samples within the analyzed sample set collected in community health care centers from symptomatic patients represented the A/H1N1/09 strain. Reports on A/H1N1/09 prevalence in 2009/2010 vary from different areas of the globe. Some indicate that while A/H1N1/09 was the predominant virus in circulation, seasonal influenza nonetheless accounted for approximately 10% of influenza cases [16]. However, our results are in accord with a report from the Centers for Disease Control and Prevention indicating that A/H1N1/09 was responsible for > 99% of influenza cases in the US between April 2009 and June 2010 [17].

It has been suggested that rhinovirus outbreaks in late summer and early fall of 2009 may have delayed the reemergence of A/H1N1/09 [18,19] through induction of innate immunity. Our survey is not inconsistent with this model. The frequency of A/H1N1/09-positive samples increased in November, after the peak activity of rhinovirus in September/October 2009. However, whether HRV infection influenced A/H1N1/09 re-appearance is unclear; late summer and early fall is the seasonal time for rhinovirus circulation and the observed pattern may thus be coincidental. However, our finding of a low co-infection rate between the two agents despite their overall high prevalence over the course of the study is compatible with an interference of rhinovirus infection with influenza.

We also identified a high frequency of enterovirus 68 infection in samples collected during September and October. Unlike other enteroviruses, enterovirus 68 has been predominately associated with respiratory disease, but is rarely reported in the US [20]. The temporal clustering of enterovirus 68 positive samples suggests a short, local outbreak.

## Conclusion

This study underscores the importance of unbiased multiplexed surveillance for the presence of respiratory pathogens. Through systematic collection of samples and the application of methods like MassTag PCR we anticipate new insights into the epidemiology of infectious diseases that will allow prioritization of investments in vaccines and drugs. We also note that one of the agents found most commonly in our samples, rhinovirus C [11-13,21], was unknown as recently as 2006, and has not yet been cultured. Given that no virus was identified in close to 50% of the specimens we examined there is clearly need for additional efforts in pathogen discovery.

## Competing interests

The authors declare that they have no competing interests.

## Authors' contributions

Conceived and designed the study: WIL, FM, TB; Coordinated sample collection: WW, JL, FM; Performed the experiments: RT, VK; Analyzed the data: RT, VK, KJ, TB; Assembled the manuscript: RT, VK, TB, WIL; All authors read, edited and approved the final manuscript.
